# Gendered sexual risk patterns and polygamy among HIV sero-discordant couples in Uganda

**DOI:** 10.1186/1742-4690-9-S1-P103

**Published:** 2012-05-25

**Authors:** Sarah Khanakwa, Moses Ngolobe, David Moore, Robert Mwesigwa, Josephine Birungi, Rachel King, Kate Shannon

**Affiliations:** 1 Aids Support Organization (Taso) Uganda, Jinja, Uganda

## Background

Multiple sexual partnerships and HIV sero-discordant relationships are among the most at-risk for HIV transmission. Polygamy is a common form of multiple-partnered relationships in Eastern Uganda. We investigated the association between HIV risk patterns and polygamy among HIV sero-discordant couples at The AIDS Support Organization in Jinja, Uganda Methods Participants were enrollees in a prospective cohort of HIV sero-discordant couples, the Highly Active Antiretroviral therapy as Prevention (HAARP) Study at TASO Jinja. Descriptive and bivariate analyses to compare sexual risk patterns among HIV sero-discordant men; in polygamous as compared to single-spouse relationship.

## Results

Polygamous Vs Single-spouse couples ≥2 wives 1 wife P value N = 241 56 185 Male HIV+ve 38 (68%) 99 (54%) 0.065 Male-controlled sexual decision making 34 (61%) 66 (36%) 0.001 Male-controlled condom use 33 (59%) 51 (28%) <0.001 Condom last time had sex 45 (80%) 128 (69%) 0.086 Financial support 45 (80%) 152 (82%) 1.00 HIV positive partner on ART 24 (37%) 88 (48%) 0.143 Median age (IQR) 44 (39– 50) 43 (37– 50) 0.451.

## Conclusion

This study demonstrates continued gendered risks for women in HIV sero-discordant relationships in sub-Saharan Africa. In particular, men with 2 or more wives are more likely to make decisions about when to have sex or when to use a condom. However, we found no differences in condom use at last sex by polygamy status.

